# The association between serum testosterone levels and metabolic syndrome among women

**DOI:** 10.1186/s13098-021-00643-6

**Published:** 2021-03-06

**Authors:** Junxiao Liang, Qiaohua Peng, Xinyun Yang, Chunbo Yang

**Affiliations:** Department of Obstetrics and Gynecology, Women’s Hospital School of Medicine Zhejiang University, No.1, Xueshi Road, Shangcheng District, Hangzhou, 310006 Zhejiang China

**Keywords:** Metabolic syndrome, Testosterone, National Health and Nutrition Examination survey (NHANES), Postmenopausal

## Abstract

**Background:**

This study aimed to investigate the relationship between total serum testosterone level (TT) and metabolic syndrome (MetS) among adult female population. Subgroup analysis further stratified the population by menopausal status to address the potential hormonal difference in postmenopausal women.

**Methods:**

A total of 1966 participants from the National Health and Nutrition Examination Survey (NHANES) 2011–2012 cycle was included for analysis in this study. MetS was defined based on the National Cholesterol Education Program Adult Treatment Panel III guidelines. Serum TT was collected during the physical examination of the NHANES program and divided into quartiles (Q) in this analysis. Menopausal status was determined based on NHANES Reproductive Health Questionnaire. Logistic regression models were applied for analysis.

**Results:**

The odds of MetS in Q2: 12.99–19.38 ng/mL (OR = 0.641, 95%CI 0.493–0.835, P < 0.01), Q3: 19.39–28.38 ng/mL (OR = 0.476, 95%CI 0.362–0.626, P < 0.001), and Q4: ≥28.40 ng/mL (OR = 0.390, 95%CI 0.294–0.517, P < 0.001) were statistically lower compared to the reference Q1: <12.99 ng/mL. For the postmenopausal group, a significantly lower odds of MetS was observed in the Q2 (OR = 0.689, 95%CI 0.486–0.977, P < 0.05) and Q4 (OR = 0.606, 95%CI 0.399–0.922, P < 0.05), while the odds of Q3 (OR = 0.439, 95%CI 0.248–0.779, P < 0.01) and Q4 (OR = 0.464, 95%CI 0.261–0.825, P < 0.01) were significantly lower than the reference Q1 in the premenopausal group.

**Conclusions:**

Elevated TT levels are associated with incremental reductions in the odds of metabolic syndrome among adult females. Although, serum testosterone level is associated with the occurrence of metabolic syndrome in both the postmenopausal and the premenopausal group, the patterns of the relationship are different.

## Background

Metabolic syndrome (MetS) is a multiplex risk factor disease characterized by abdominal obesity, insulin resistance, hypertension, and dyslipidemia [1]. In the United States, the prevalence of MetS has increased substantially from 1988 to 2012, rising from 25.29 to 34.17% of the adult population according to the National Health and Nutrition Examination Survey (NHANES) data [2]. Serum testosterone levels (TT) have long been associated with the development and incidence of MetS or its component [3–6]. It has been proposed that TT may influence the development of MetS by increasing skeletal muscle tissue and decreasing abdominal obesity and non-esterified fatty acids [7].

Although the association between TT and MetS of the adult male population has been comprehensively studied in previous studies, indicating that low concentration of TT level is associated with a higher prevalence of MetS [3–6], such association of the female population has not been fully revealed. The TT production of females is interrupted under certain conditions, such as menopause. At the menopausal stage, the depletion of oocytes and ovarian follicles is accompanied by the decrease in ovarian androgen production, which subsequently leads to a decline in the TT levels [8]. Therefore, the decreased TT level at menopause may influence the development of MetS.

To our knowledge, only a few researches have examined the association between TT and MetS of the female population [9–11]. Thus, this study aimed to investigate the relationship between TT levels and MetS of the adult female population and further, to compare the TT level and the elements of MetS between postmenopausal and premenopausal women.

## Methods

This cross-sectional study analyzed data from the 2011–2012 National Health and Nutrition Examination Survey (NHANES) cycle. The Center for Disease Prevention and Control has conducted the NHANES program since the 1960s, aiming to provide nationally-representative vital health statistics. In each of the two-year cycle, the NHANES recruited approximately 10,000 non-institutionalized civilian resident population in the United States to collect health and nutritional status through home interviews and physical examinations in the Mobile Examination Center (MEC). All NHANES data collection protocols were approved by the National Center of Health Statistics Research Ethics Review Board [12]. This study analyzed de-identified downloadable public NHANES data and was exempt from future ethics review board approval of Women’s Hospital School of Medicine Zhejiang University.

### Study participants

In total, 9756 women participated in the NHANES 2011–2012 cycle. Since this study tended to explore the association between TT level and MetS among the adult female population, male participants, and participants who reported less than 20 years old were excluded. Pregnant and breastfeeding participants were subject to the shift in hormone level as well as the effect on the metabolism and were thus excluded in this study [13, 14]. Demographic characteristics included age, body mass index (BMI), and race.

### Diagnosis of metabolic syndrome

The diagnosis of MetS was determined based on the National Cholesterol Education Program Adult Treatment Panel III guidelines [15]. The components of the MetS diagnosis include (1) Elevated waist circumference (> 88 cm in women), (2) Elevated serum triglycerides (≥ 150 mg/dL), (3) Reduced HDL-C (< 50 mg/dL in women), (4) High Blood pressure (≥ 130/85 mm Hg), (5) Elevated serum glucose (≥ 110 mg/dL) [16]. The study participants were categorized in the MetS group if three or more of the above diagnostic abnormities were fulfilled.

### Serum testosterone measurement

Biospecimens, including blood, urine, oral rinse, and vaginal swabs, were collected during the MEC examination for laboratory analysis. The blood sample was used to assess the testosterone level as well as the indicators of MetS. The NHANES collected serum TT as an indicator of the testosterone level. Since the bound and unbound testosterone level were not separated in the database, this research used TT as an evaluation of the testosterone level. As indicated in the NHANES Laboratory Procedure Manual, routine quantitation of TT was based on the National Institute for Standards and Technology’s reference method, which is a validated isotope dilution liquid chromatography-tandem mass spectrometry (ID-LC-MS/MS) method [17].

### Menopause status

Menopausal status was assessed based on the NHANES Reproductive Health Questionnaire, where RHD042 asked, “What is the reason that {you have/SP has} not had a period in the past 12 months?” Participants who answered “Menopause” or “Hysterectomy” were categorized as the postmenopausal group.

### Statistical analysis

The demographic characteristics of the study population were compared using independent samples t-test, Pearson’s chi-square test (χ^2^), and Fisher’s exact test when appropriate. Continuous variables were tested for normality using the Shapiro-Wilk test. Mann-Whitney U test was applied to compare variables with abnormal distribution. Summary statistics presented the mean and standard deviation of the continuous variables (Mean ± SD), and frequencies and percent distributions of categorical variables (N%). Both unadjusted and adjusted logistic regression models were used to acquire the Odds Ratio (OR), 95% Confidence Interval (CI), and statistical significance of MetS and each component in the overall population. Additionally, subgroup analyses were performed to investigate both the postmenopausal and premenopausal groups using logistic regression models similar to the overall population analyses. The NHANES cycle oversampled a certain group of minorities to increase the representativeness of the sample. In the 2011–2012 survey year, the oversample domains were non-Hispanic black persons, non-Hispanic non-black Asian persons, Hispanic persons, and low- and non-low-income groups. The full sample 2-year MEC examination weight (WTMEC2YR) found in the NHAENS database was applied to all analyses in this study. All statistical analyses were performed using SPSS Statistics 23.0 (IBM Corporation. Armonk, NY, USA) and SAS 9.4 (SAS Institute, inc. Cary, NC, USA). A *P* value of less than 0.05 is defined as significant.

## Results

### Study population

After excluding males (n = 4856), participants aged less than 20 years old (n = 2080), pregnancy and breastfeeding (n = 10), and participants with missing data in age, sex, BMI, race, menopausal status, and serum TT level (n = 844), a total of 1966 participants were included in the analysis of this study. Of the included 1966 participants, the average age was 49 ± 17 years old, and the average BMI was 29.38 ± 7.33 kg/m^2^. Ratio distribution showed that 19.94% of the participants were Hispanic, 38.10% were non-Hispanic white, 27.31% were black, 12.21% were Asian, and 2.44% were others. The sample is balanced for menopausal status, with 50.05% and 49.95% of the participants classified as the premenopausal and the postmenopausal groups. Serum TT level of the overall population was divided into quartiles (Q), which ranged from < 12.99 ng/mL in Q1, 12.99–19.38 ng/mL in Q2, 19.39–28.38 ng/mL in Q3, and ≥ 28.40 ng/mL in Q4. There were 571 women (28.6%) categorized in the MetS group and 1,395 (71.39%) women in the non-MetS group.

### Demographics

When comparing the MetS group and the non-MetS group (referred to the control group), age, BMI, race, menopausal status, and TT levels were significantly different (Table 1).

**Table 1:** Demographic characteristics of study participants in the NHANES 2011–2012

Variables	Total (n = 1966) N%^b^	MetS^a^ (n = 571) N%	Non-MetS (n = 1395) N%	*P*^c^
Age (years), n (%)				< 0.001
20–39	670 (34.08)	98 (17.16)	572 (41.00)	
40–49	316 (16.07)	92 (16.11)	224 (16.06)	
50–59	336 (17.09)	126 (22.07)	210 (15.05)	
60–69	348 (17.70)	129 (22.59)	219 (15.70)	
≥ 70	296 (15.06)	126 (22.07)	170 (12.19)	
BMI (kg/m^2^), n (%)				< 0.001
< 18.5	43 (2.19)	2 (0.35)	41 (2.94)	
18.5–24.9	572 (29.09)	50 (8.76)	522 (37.42)	
25.0-29.9	556 (28.28)	156 (27.32)	400 (28.67)	
≥ 30.0	795 (40.44)	363 (63.57)	432 (30.97)	
Race, n (%)				< 0.001
Hispanic	392 (19.94)	134 (23.47)	258 (18.49)	
Non-hispanic white	749 (38.10)	230 (40.28)	519 (37.20)	
Black	537 (27.31)	157 (27.50)	380 (27.24)	
Asian	240 (12.21)	46 (8.06)	194 (13.91)	
Other	48 (2.44)	4 (0.70)	44 (3.15)	
Menopausal status, n (%)				< 0.001
No	984 (50.05)	189 (33.10)	795 (56.99)	
Yes	982 (49.95)	382 (66.90)	600 (43.01)	
Serum testosterone (ng/mL), n (%)				< 0.001
Q_1_ (< 12.99)	492 (25.03)	199 (34.85)	293 (21.00)	
Q_2_ (12.99–19.38)	491 (24.97)	149 (26.09)	342 (24.52)	
Q_3_ (19.39–28.38)	491 (24.97)	120 (21.02)	371 (26.59)	
Q_4_ (≥ 28.40)	492 (25.03)	103 (18.04)	389 (27.89)	

^a^MetS: metabolic syndrome group

^b^N%: frequency and percentage of the total

^c^*P* value: Results from Pearson’s chi-square test and Fisher’s exact test

The percentage of the mid-aged individual (50–59 years old) and elders (60–69 and 70 years old) were higher in the MetS group than the control group (Z = 10.38, *P* < 0.001). The MetS group indicated higher rates of overweight (27.32% vs. 28.67) and obese (63.57% vs. 30.97%) than that of the control group (Z = 15.23, *P* < 0.001). Higher percentages of Hispanic and Non-Hispanic white participants were discovered in the MetS group than the control group (χ^2^ = 27.39, *P* < 0.001). The menopausal status of the MetS group and the control group were statistically different (χ^2^ = 92.49, *P* < 0.001), with the MetS group illustrating a higher proportion of participants classified as postmenopausal than the control group (66.9% vs. 43.1%). Additionally, the distribution of TT level was different between the MetS and control group, with more participants falling in the lowest quartile (34.85% vs. 21%) and the second quartile (26.9% vs. 24.52%) than the control group (Z = − 7.04, *P* < 0.001).

### Serum testosterone

In all the unadjusted and adjusted logistic regression models, the TT level in the first quartile (< 12.99 ng/mL) was the reference group. For the overall population, participants in Q2 (OR = 0.641, 95%CI 0.493–0.835, *P* < 0.01), Q3 (OR = 0.476, 95%CI 0.362–0.626, *P* < 0.001), and Q4 (OR = 0.390, 95%CI 0.294–0.517, *P* < 0.001) showed a statistically significant lower odds of MetS when compared to Q1 (Table 2).

**Table 2:** Association of serum testosterone level and metabolic syndrome assessed by logistic regression models

Overall population				
Serum testosterone level (ng/mL)	Unadjusted		Adjusted^a^	
	OR [95CI]^b^	*P*^c^	OR [95CI]	*P*
Metabolic syndrome				
Q_1_ (< 12.99)	Ref		Ref	
Q_2_ (12.99–19.38)	0.641 [0.493–0.835]	0.01	0.687 [0.511–0.924]	0.05
Q_3_ (19.39–28.38)	0.476 [0.362–0.626]	0.001	0.575 [0.421–0.784]	0.001
Q_4_ (≥ 28.40)	0.390 [0.294–0.517]	0.001	0.536 [0.386–0.745]	0.001
Elevated waist circumference (> 88 cm)				
Q_1_ (< 12.99)	Ref		Ref	
Q_2_ (12.99–19.38)	0.766 [0.582–1.008]		0.923 [0.585–1.456]	
Q_3_ (19.39–28.38)	0.699 [0.532–0.918]	0.05	0.898 [0.553–1.459]	
Q_4_ (≥ 28.40)	0.647 [0.493–0.848]	0.01	1.005 [0.611–1.651]	
Elevated serum triglycerides (≥ 150 mg/dL)				
Q_1_ (< 12.99)	Ref		Ref	
Q_2_ (12.99–19.38)	0.648 [0.500-0.841]	0.001	0.651 [0.493–0.860]	0.01
Q_3_ (19.39–28.38)	0.472 [0.361–0.619]	0.001	0.541 [0.404–0.725]	0.001
Q_4_ (≥ 28.40)	0.372 [0.281–0.492]	0.001	0.488 [0.357–0.666]	0.001
Reduced HDL-C (< 50 mg/dL)				
Q_1_ (< 12.99)	Ref		Ref	
Q_2_ (12.99–19.38)	0.805 [0.620–1.044]		0.717 [0.543–0.948]	0.05
Q_3_ (19.39–28.38)	0.769 [0.592–0.999]	0.05	0.618 [0.464–0.824]	0.01
Q_4_ (≥ 28.40)	0.671 [0.515–0.875]	0.01	0.520 [0.384–0.703]	0.001
Elevated blood pressure (≥ 130/85 mm Hg)				
Q_1_ (< 12.99)	Ref		Ref	
Q_2_ (12.99–19.38)	0.646 [0.498–0.836]	0.01	0.932 [0.699–1.243]	
Q_3_ (19.39–28.38)	0.468 [0.358–0.612]	0.001	0.854 [0.630–1.159]	
Q_4_ (≥ 28.40)	0.392 [0.298–0.516]	0.001	0.814 [0.588–1.127]	
Elevated serum glucose level (≥ 110 mg/dL)				
Q_1_ (< 12.99)	Ref		Ref	
Q_2_ (12.99–19.38)	0.675 [0.500-0.912]	0.05	0.771 [0.561–1.060]	
Q_3_ (19.39–28.38)	0.564 [0.413–0.770]	0.001	0.744 [0.532–1.039]	
Q_4_ (≥ 28.40)	0.354 [0.251-0.500]	0.001	0.525 [0.361–0.763]	0.001

^a^ Adjusted: Logistic regression model adjusted for Age, BMI, Race, and Menopausal Status

^b^OR [95CI]: Odds Ratio [95% Confident Interval]

^c^*P* value: Results from logistic regression models, including statistically significant results exclusively

After adjusting for age, BMI, race, and menopausal status, the relationship remained consistent, with all three quartiles, Q2 (OR = 0.687, 95%CI 0.511–0.924, *P* < 0.05), Q3 (OR = 0.575, 95%CI 0.419–0.778, *P* < 0.001), and Q4 (OR = 0.536, 95%CI 0.386–0.745, *P* < 0.001), demonstrating a significant decrease in the occurrence of MetS. When analyzing the waist circumference, participants in Q3 (OR = 0.699, 95%CI 0.532–0.918, *P* < 0.05) and Q4 (OR = 0.647, 95%CI 0.493–0.848, *P* < 0.01) indicated a significantly lower odds of elevated waist circumference (> 88 cm) than the reference group in the unadjusted model. However, the significance did not retain after controlling for age, BMI, race, and menopausal status. Similarly, a statistically lower occurrence of elevated blood pressure was observed across all quartiles of the unadjusted model, but not in the adjusted logistic regression model.

The odds of having elevated serum triglyceride (≥ 150 mg/dL) significantly declined in all three quartiles of the unadjusted model, Q2 (OR = 0.648, 95%CI 0.500-0.841, *P* < 0.001), Q3(OR = 0.472, 95%CI 0.361–0.619, *P* < 0.001), and Q4 (OR = 0.372, 95%CI 0.281–0.492, *P* < 0.001), as well as the adjusted model, Q2 (OR = 0.651, 95%CI 0.493–0.860, *P* < 0.001), Q3 (OR = 0.541, 95%CI 0.404–0.725, *P* < 0.01), and Q4 (OR = 0.488, 95%CI 0.357–0.666, *P* < 0.001). Decreased occurrence of low HDL-C (< 50 mg/dL) was detected among participants in Q2 (OR = 0.769, 95%CI 0.592–0.999, *P* < 0.05) and Q3 (OR = 0.671, 95%CI 0.515–0.875, *P* < 0.01) in the unadjusted model, while the Q2 (OR = 0.717, 95%CI 0.543–0.948, *P* < 0.05), Q3(OR = 0.618, 95%CI 0.464–0.824, *P* < 0.01), and Q4 (OR = 0.520, 95%CI 0.384–0.703, *P* < 0.001) in the adjusted model demonstrated an incremental reduction in the odds of low HDL-C. When analyzing the odds of high serum glucose (≥ 110 mg/dL) using the unadjusted model, the odds in Q2 (OR = 0.675, 95%CI 0.500–0.912, *P* < 0.05), Q3 (OR = 0.564, 95%CI 0.413–0.770, *P* < 0.001), and Q4 (OR = 0.354, 95%CI 0.251–0.500, *P* < 0.001) were significantly decreased as compared to Q1. However, only Q4 (OR = 0.525, 95%CI 0.361–0.763, *P* < 0.001) was found to decrease when controlling for covariates.

Subgroup analysis was conducted to explore the effect of TT on MetS and its components in both the postmenopausal (n = 984) and premenopausal (n = 982) groups, as summarized in Table 3. In the postmenopausal group, a significantly lower odds of MetS was observed among participants of the Q4 when applying the unadjusted logistic regression model (OR = 0.655, 95%CI 0.443–0.970, *P* < 0.05). In the logistic regression model adjusting for age, BMI, and race, both participants in Q2 (OR = 0.689, 95%CI 0.486–0.977, *P* < 0.05) and Q4 (OR = 0.606, 95%CI 0.399–0.922, *P* < 0.05) indicated significantly lower odds of MetS.

**Table 3:** Association of serum testosterone level and metabolic syndrome stratified by menopausal status

Serum testosterone level (ng/mL)	Postmenopausal				Premenopausal			
	Unadjusted		Adjusted^a^		Unadjusted		Adjusted	
	OR [95CI]^b^	*P*^c^	OR [95CI]	*P*	OR [95CI]	*P*	OR [95CI]	*P*
Metabolic syndrome								
Q_1_ (< 12.99)	Ref		Ref		Ref		Ref	
Q_2_ (12.99–19.38)	0.743 [0.537–1.029]		0.689 [0.486–0.977]	0.05	0.678 [0.416–1.107]		0.739 [0.417–1.309]	
Q_3_ (19.39–28.38)	0.787 [0.552–1.122]		0.701 [0.480–1.023]		0.408 [0.249–0.670]	0.001	0.439 [0.248–0.779]	0.01
Q_4_ (≥ 28.40)	0.655 [0.443–0.970]	0.05	0.606 [0.399–0.922]	0.05	0.394 [0.243–0.640]	0.001	0.464 [0.261–0.825]	0.01
Elevated waist circumference (> 88 cm)								
Q_1_ (< 12.99)	Ref		Ref		Ref		Ref	
Q_2_ (12.99–19.38)	1.010 [0.698–1.462]		0.982 [0.558–1.728]		0.733 [0.464–1.156]		0.823 [0.367–1.843]	
Q_3_ (19.39–28.38)	1.144 [0.756–1.729]		0.809 [0.422–1.552]		0.704 [0.454–1.092]		0.919 [0.411–2.054]	
Q_4_ (≥ 28.40)	1.188 [0.756–1.867]		1.108 [0.552–2.223]		0.695 [0.452–1.070]		0.916 [0.409–2.050]	
Elevated serum triglycerides (≥ 150 mg/dL)								
Q_1_ (< 12.99)	Ref		Ref		Ref		Ref	
Q_2_ (12.99–19.38)	0.755 [0.547–1.043]		0.679 [0.485–0.952]	0.05	0.657 [0.408–1.058]		0.672 [0.404–1.120]	
Q_3_ (19.39–28.38)	0.631 [0.441–0.904]	0.05	0.581 [0.399–0.845]	0.01	0.486 [0.304–0.777]	0.01	0.560 [0.338–0.928]	0.05
Q_4_ (≥ 28.40)	0.368 [0.240–0.563]	0.01	0.381 [0.244–0.592]	0.001	0.499 [0.316–0.789]	0.01	0.622 [0.376–1.030]	
Reduced HDL-C (< 50 mg/dL)								
Q_1_ (< 12.99)	Ref		Ref		Ref		Ref	
Q_2_ (12.99–19.38)	0.724 [0.514–1.017]		0.656 [0.459–0.938]	0.05	0.733 [0.471–1.142]		0.815 [0.503–1.322]	
Q_3_ (19.39–28.38)	0.854 [0.592–1.231]		0.788 [0.538–1.153]		0.559 [0.363–0.859]	0.01	0.497 [0.309–0.801]	0.01
Q_4_ (≥ 28.40)	0.613 [0.403–0.930]	0.05	0.620 [0.401–0.959]	0.05	0.531 [0.347–0.810]	0.01	0.442 [0.273–0.714]	0.001
Elevated serum glucose level (≥ 110 mg/dL)								
Q_1_ (< 12.99)	Ref		Ref		Ref		Ref	
Q_2_ (12.99–19.38)	0.839 [0.611–1.152]		0.926 [0.663–1.293]		0.928 [0.522–1.651]		0.962 [0.526–1.757]	
Q_3_ (19.39–28.38)	0.803 [0.568–1.137]		0.831 [0.576–1.199]		0.692 [0.391–1.226]		0.890 [0.486–1.631]	
Q_4_ (≥ 28.40)	0.989 [0.679–1.439]		0.829 [0.557–1.234]		0.510 [0.285–0.913]	0.05	0.793 [0.422–1.489]	

^a^Adjusted: Logistic regression model adjusted for Age, BMI, and Race

^b^OR [95CI]: Odds Ratio [95% Confident Interval]

^c^*P* value: Results from logistic regression models, including statistically significant results exclusively

In the premenopausal group, the results were consistent in both the unadjusted and adjusted logistic regression models. In the unadjusted model, the odds of Q3 (OR = 0.408, 95%CI 0.249–0.670, *P* < 0.001) and Q4 (OR = 0.394, 95%CI 0.243–0.640, *P* < 0.001) were significantly lower than Q1. Similarly, the odds of Q3 (OR = 0.439, 95%CI 0.248–0.779, *P* < 0.01) and Q4 (OR = 0.464, 95%CI 0.261–0.825) were significantly lower than Q1 when adjusting for age, BMI, and race.

When investigating the odds of elevated waist circumference (> 88 cm), no statistical difference was found across all quartiles when compared to the reference, which remained consistent in both the postmenopausal and premenopausal group. In the postmenopausal group, a significantly reduced occurrence of elevated serum triglycerides (≥ 150 mg/dL) was detected in Q3 (OR = 0.631, 95%CI 0.441–0.904, *P* < 0.05)and Q4 (OR = 0.368, 95%CI 0.240–0.563, *P* < 0.001) of the unadjusted model, while significance was detected in all Q2 (OR = 0.679, 95%CI 0.485–0.952, *P* < 0.05), Q3 (OR = 0.581, 95%CI 0.399–0.845, *P* < 0.01) and Q4 (OR = 0.381, 95%CI 0.244–0.592, *P* < 0.001) when controlling for age, BMI, and race. Similarly, a reduction was also observed in the Q3 (OR = 0.486, 95%CI 0.304–0.777, *P* < 0.01) and Q4 (OR = 0.499, 95%CI:0.316–0.789, *P* < 0.01) in the uncontrolled analysis of the premenopausal group. After adjusting for age, BMI, and race, only the third quartile Q3 (OR = 0.560, 95%CI 0.338–0.928, *P* < 0.05) showed a reduction in high serum triglyceride odds.

Comparing to the reference group, the Q4 (OR = 0.613, 95%CI 0.403–0.930, *P* < 0.05) of the postmenopausal group showed a lower OR of reduced HDL-C (< 50 mg/dL) using the unadjusted model, while both Q2 (OR = 0.656, 95%CI 0.459–0.938, *P* < 0.05) and Q4 (OR = 0.620, 95%CI 0.401–0.959, *P* < 0.05) showed a decreased OR after adjusting for age, BMI, and race. In the premenopausal group, a reduced occurrence of low HDL-C level was detected in both the Q3 (OR = 0.559, 95%CI 0.363–0.859, *P* < 0.01) and Q4 (OR = 0.531, 95%CI 0.347–0.810, *P* < 0.01) of the uncontrolled model and the Q3 (OR = 0.497, 95%CI 0.309–0.801, *P* < 0.01) and Q4 (OR = 0.442, 95%CI 0.273–0.714, *P* < 0.01) of the controlled model.

No difference in the odds of high blood pressure was discovered in the postmenopausal group. In the premenopausal group, only the OR of Q4 (OR = 0.510, 95%CI 0.285–0.913, *P* < 0.05) was significantly lower than the reference group in the unadjusted model. For the high serum glucose, the odds of all three quartiles were not statistically different from the reference in the postmenopausal group. However, discrepancies were detected in the unadjusted model of the premenopausal group, with Q3 (OR = 0.481, 95%CI 0.274–0.843, *P* < 0.05) and Q4 (OR = 0.221, 95%CI 0.117–0.416, *P* < 0.001) odds being significantly lower than the reference group. When adjusting for age, BMI, and race, the odds of Q3 (OR = 0.548, 95%CI 0.301–0.998, *P* < 0.05) and Q4 (OR = 0.286, 95%CI 0.145–0.565, *P* < 0.001) were significantly lower than the reference.

## Discussion

Aiming to evaluate whether an association exists between the serum TT and metabolic syndrome (MetS) in the adult female population, this study analyzed data from the 2011–2012 NHANES. Age, BMI, race, and menopausal status are associated with MetS [4, 18, 19], and are therefore compared as potential covariates between the MetS and non-MetS groups. Since all potential confounding variables were statistically different between the MetS and non-MetS groups in the demographic comparison, these covariates were later controlled in the adjusted logistic regression model in the present study.

As the TT level increased, we detected an incremental reduction in the odds of MetS independently from age, BMI, race, and menopausal status. When comparing to the reference TT level (< 12.99 ng/mL), the TT level at ≥ 28.4 ng/mL indicated the most decrease in the OR, followed by the TT level at 19.39–28.38 ng/mL and TT level at 12.99–19.38 ng/mL, corresponding to 46.4%, 42.5%, and 31.3% reduction in the occurrence of MetS. The inverse relationship between TT level and MetS discovered in our study is in accordance with previous observational studies in the male population [3, 4, 6, 20–23].

In addition to the odds of MetS, we analyzed the impact of TT on the components of MetS to consider the underlying mechanisms of TT on the development of MetS in the female population. Interestingly, TT level was only associated with certain components of the MetS, including serum triglycerides, HDL-C, and serum glucose. Decreased odds of high serum triglyceride level and low HDL-C level were detected in an incremental pattern. TT level at 12.99–19.38 ng/mL, 19.39–28.38 ng/mL, and ≥ 28.40 ng/mL corresponded to a 34.9%, 45.9%, and 51.2% decline in the occurrence of elevated serum triglyceride. Similarly, a 28.3%, 38.2%, and 48% reduction in the odds of low HDL-C level was found at TT level 12.99–19.38 ng/mL, 19.39–28.38 ng/mL, and ≥ 28.40 ng/mL, respectively. For high serum glucose levels, only the highest TT level showed a significant decrease (47.5%) in the OR compared to the reference.

The results of the present study indicate that higher serum TT ameliorates lipid and glucose metabolism-related MetS components more profoundly than other components, such as hypertension and waist circumference, in the female population. Nevertheless, previous observational studies in the male population revealed that TT level was correlated with all MetS components, including reduced total cholesterol, reduced systolic and diastolic blood pressure, lower blood glucose, and lower waist circumference [20, 24, 25]. Gender disparities of human blood pressure regulation may contribute to the discrepancy in the odds of high blood pressure. Hypertension was more prevalent in men than women, and the rise of blood pressure with age was slower in women than men [26]. Furthermore, variations in the sample size of different studies may also contribute to the discrepancies in the results.

To address the potential hormonal disparities between postmenopausal and premenopausal participants, subgroup analysis stratified by menopausal status was performed in this research. The postmenopausal and premenopausal groups are associated with MetS odds as the TT level elevates. In the postmenopausal group, a decline in the odds of MetS was discovered at both TT level 12.99–19.38 ng/mL (31.1%) and ≥ 28.40 ng/mL (39.4%.), while no significant decrease in OR was detected at TT level 19.39–28.38 ng/mL. When investigating the components of MetS in the postmenopausal group, only high serum triglyceride and low HDL-C were related to the increase of TT level. TT level at 12.99–19.38 ng/mL, 19.39–28.38 ng/mL, and ≥ 28.40 ng/mL resulted in 32.1%, 41.9%, 61.9% reduction in the occurrence of high serum triglyceride. The odds of decreased HDL-C were 34.4% and 38% lowered when the TT level increased to 12.99–19.38 ng/mL and ≥ 28.40 ng/mL as compared to the lowest TT level.

In the premenopausal group, TT level at 19.39–28.38 ng/mL and ≥ 28.40 ng/mL were related to lower occurrences of MetS. However, the reduction tended to be decremental, with the third quartile illustrating a 56.1% reduction and the fourth quartile demonstrating a 53.6% decrease. Elevated serum triglycerides, reduced HDL-C, and high serum glucose were associated with the increasing TT level in the premenopausal group. The occurrence of high serum triglycerides was 44.0% lower than the reference at TT level 12.99–19.38 ng/mL but not at other TT levels. TT level at 19.39–28.38 ng/mL and ≥ 28.40 ng/mL led to a 50.3% and 55.8% incremental reduction in the odds of decreased HDL-C level, as well as a 45.2% and 71.4% increase in the occurrence of high serum glucose.

Although TT serves as an independent predictor of MetS in both the postmenopausal and premenopausal groups, the findings of this research demonstrate disparities in the patterns and odds of the MetS components, as well as odds of low HDL-C level. Additionally, TT is a stronger predictor of the odds of high serum triglycerides in the postmenopausal group as compared to the premenopausal group. Conversely, a more potent relationship exists between TT and the odds of hyperglycemia in the premenopausal group when comparing to the postmenopausal group.

The analysis of the components of the MetS uncovers a more detailed impact of TT on MetS. Furthermore, we use population-based nationally representative data from the NHANES database, making the results of this research generalizable to the U.S. population. To our knowledge, this is the first study that uses generalizable large sample to analyze the relationship between TT level and MetS. Although a correlation is unearthed, this observational study cannot establish a causal relationship between the predictor and the outcome. Hence, the direction of the relationship between TT level and MetS is not ascertained in our research. Notably, research has provided compelling evidence that MetS patients are predisposed to lower TT levels due to hyperglycemia and obesity [27, 28], implying a bidirectional relationship between MetS and TT level. Another limitation is that the NHANES data did not separate participants who were menopausal and who underwent hysterectomy during data collection. The postmenopausal group in the current study combined menopausal participants and hysterectomy participants. However, the risk of metabolic outcomes after hysterectomy is only slightly higher than that of the non-hysterectomy group, from 1.13 to 1.33 times higher [29]. Therefore, combining women who underwent hysterectomy and women who did not undergo hysterectomy does not impose a significant impact on the outcomes variables and validity of the results.

For males, the TT level below 300 ng/dL is considered low, according to the American Urologic Association [30]. However, the reference TT levels of adult females are not consistent because of the divergent considerations of human development and health risks. Although our results suggest TT level is inversely associated with the MetS events, an adverse impact may appear when the TT level elevates to a superfluous degree in women. For instance, polycystic ovary syndrome (PCOS), a reproductive disorder characterized by hyperandrogenism, polycystic ovaries, and anovulation, has been found to comorbid with MetS [31, 32], illustrating a negative influence of excess TT on MetS.

Moreover, Sex hormones-binding globulin (SHBG) and human serum albumin binding protein, the testosterone cognate binding proteins, are essential in regulating circulating testosterone distribution [33]. Testosterone that is unbound to any plasma binding protein is referred to free testosterone, which reflects clinical condition more accurately than TT due to its bioavailability [34]. In this research, we applied TT as the predictor in the analysis because the bound and unbound testosterone were inseparable using the NHANES database. Therefore, future studies aiming to evaluate the correlation between serum testosterone level and the risk of MetS may separate bioavailable testosterone from TT to acquire a more comprehensive interpretation of the effect.

The efficacy of testosterone therapy in ameliorating the components of MetS has been justified in multiple studies of the male population [24, 25]. Nevertheless, testosterone replacement therapy is not recommended for the female population to mitigate MetS due to the insufficient understanding and evidence in supporting testosterone therapy on females. Moreover, the consequence of low TT level on women has long been underestimated, resulting in the lack of concrete guidelines in defining testosterone deficiency among women. Overall, our study provides a preliminary insight into the impact of low testosterone levels on premenopausal and postmenopausal women, which may be derived to elucidate potential hormonal therapies for women in preventing and managing MetS.

## Conclusions

Our findings show the permissive role of serum testosterone in the pathogenesis of MetS in the adult female population. In this research, an increased serum TT level is accompanied by an incremental decrease in the odds of MetS, with TT level at ≥ 28.40 ng/mL showing the greatest reduction, 46.40%, as compared to TT level at < 12.99 ng/mL. Two components of the MetS, elevated serum triglyceride and decreased HDL-C level, are strongly associated with the rising of TT. Serum TT levels affect MetS events in both postmenopausal and premenopausal groups, but the patterns are different between the two groups. Future studies are necessary to determine the optimal range of TT level for women to minimize the development of MetS and capture more integrated mechanisms for clinical implications.

## Data Availability

The datasets used and analyzed during the current study are available from the corresponding author on reasonable request.

## Abbreviations

TT: Total testosterone
MetS: Metabolic syndrome
NHANES: Nutrition Examination Survey
Q: Quartiles
MEC: Mobile Examination Center
ID-LC-MS/MS: Isotope dilution liquid chromatography-tandem mass spectrometry
BMI: Body mass index

## References

[1] Metabolic Syndrome | NHLBI, NIH. https://www.nhlbi.nih.gov/health-topics/metabolic-syndrome. Accessed 9 Oct 2020.

[2] Moore JX, Chaudhary N, Akinyemiju T (2017). Metabolic syndrome prevalence by race/ethnicity and sex in the united states, national health and nutrition examination survey, 1988–2012. Prev Chronic Dis.

[3] Brand JS, Rovers MM, Yeap BB, Schneider HJ, Tuomainen T-P, Haring R (2014). Testosterone, sex hormone-binding globulin and the metabolic syndrome in men: an individual participant data meta-analysis of observational studies. PLoS ONE.

[4] Rodriguez A, Muller DC, Metter EJ, Maggio M, Harman SM, Blackman MR (2007). Aging, androgens, and the metabolic syndrome in a longitudinal study of aging. J Clin Endocrinol Metab.

[5] Corona G, Monami M, Rastrelli G, Aversa A, Tishova Y, Saad F (2011). Testosterone and metabolic syndrome: a meta-analysis study. J Sex Med.

[6] Stellato RK, Feldman HA, Hamdy O, Horton ES, McKinlay JB (2000). Testosterone, sex hormone-binding globulin, and the development of type 2 diabetes in middle-aged men: prospective results from the Massachusetts male aging study. Diabetes Care.

[7] Laaksonen DE, Niskanen L, Punnonen K, Nyyssönen K, Tuomainen T-P, Valkonen V-P (2004). Testosterone and sex hormone–binding globulin predict the metabolic syndrome and diabetes in middle-aged men. Diabetes Care.

[8] Sarrel PM (2002). Androgen deficiency: menopause and estrogen-related factors. Fertil Steril.

[9] Ziaei S, Mohseni H (2013). Correlation between hormonal statuses and metabolic syndrome in postmenopausal women. J Fam Reprod Health.

[10] Patel SM, Ratcliffe SJ, Reilly MP, Weinstein R, Bhasin S, Blackman MR (2009). Higher serum testosterone concentration in older women is associated with insulin resistance, metabolic syndrome, and cardiovascular disease. J Clin Endocrinol Metab.

[11] Janssen I, Powell LH, Crawford S, Lasley B, Sutton-Tyrrell K (2008). Menopause and the Metabolic Syndrome. Arch Intern Med..

[12] NHANES - about the national health and nutrition examination survey. Published January 8, 2020. https://www.cdc.gov/nchs/nhanes/about_nhanes.htm. Accessed 13 Oct 2020.

[13] Schock H, Zeleniuch-Jacquotte A, Lundin E, Grankvist K, Lakso H-Å, Idahl A (2016). Hormone concentrations throughout uncomplicated pregnancies: a longitudinal study. BMC Pregnancy Childbirth.

[14] Teixeira HCA, Barbosa EA, Souto PLG, Mariante A, Ramos S (2017). Postpartum hormone and energy profiles and their influence on the resumption of ovarian cyclicity in Curraleiro Pé-Duro cows. Theriogenology.

[15] Cleeman J. ATP III guidelines At-A-glance quick desk reference. Published May 6, 2001. https://www.nhlbi.nih.gov/files/docs/guidelines/atglance.pdf. Accessed9 Oct 2020.

[16] Grundy Scott M, Cleeman James I, Daniels Stephen R, Donato Karen A, Eckel Robert H, Franklin Barry A (2005). Diagnosis and management of the metabolic syndrome. Circulation..

[17] NHANES 2011-2012: total testosterone data documentation, codebook, and frequencies. https://www.cdc.gov/Nchs/Nhanes/2011-2012/TST_G.htm. Accessed 14 Oct 2020.

[18] Patni R, Mahajan A (2018). The metabolic syndrome and menopause. J -Life Health.

[19] Moore JX, Chaudhary N, Akinyemiju T (2017). Metabolic syndrome prevalence by race/ethnicity and sex in the united states, national health and nutrition examination survey, 1988–2012. Prev Chronic Dis.

[20] Kim M, Kyung YS, Ahn TY (2020). Cross-Sectional association of metabolic syndrome and its components with serum testosterone levels in a korean-screened population. World J Mens Health..

[21] Laaksonen DE, Niskanen L, Punnonen K, Nyyssonen K, Tuomainen TP, Salonen R (2003). Sex hormones, inflammation and the metabolic syndrome: a population-based study. Eur J Endocrinol..

[22] Laaksonen DE, Niskanen L, Punnonen K, Nyyssönen K, Tuomainen T-P, Valkonen V-P (2004). Testosterone and sex hormone-binding globulin predict the metabolic syndrome and diabetes in middle-aged men. Diabetes Care.

[23] Muller M, Grobbee DE, den Tonkelaar I, Lamberts SWJ, van der Schouw YT (2005). Endogenous sex hormones and metabolic syndrome in aging men. J Clin Endocrinol Metab..

[24] Traish AM, Haider A, Doros G, Saad F (2014). Long-term testosterone therapy in hypogonadal men ameliorates elements of the metabolic syndrome: an observational, long-term registry study. Int J Clin Pract.

[25] Yassin D-J, Doros G, Hammerer PG, Yassin AA (2014). Long-term testosterone treatment in elderly men with hypogonadism and erectile dysfunction reduces obesity parameters and improves metabolic syndrome and health-related quality of life. J Sex Med..

[26] Joyner MJ, Wallin BG, Charkoudian N (2016). Sex differences and blood pressure regulation in humans. Exp Physiol.

[27] Bellastella G, Menafra D, Puliani G, Colao A, Savastano S (2019). How much does obesity affect the male reproductive function?. Int J Obes Suppl.

[28] Iranmanesh A, Lawson D, Veldhuis JD (2012). Glucose ingestion acutely lowers pulsatile LH and basal testosterone secretion in men. Am J Physiol-Endocrinol Metab..

[29] Laughlin-Tommaso SK, Khan Z, Weaver AL, Smith CY, Rocca WA, Stewart EA (2018). Cardiovascular and metabolic morbidity after hysterectomy with ovarian conservation: a cohort study. Menopause N Y N.

[30] American Urological Association. Evaluation and management of testosterone deficiency. (2018). Testosterone Deficiency Guideline; 2018. https://www.auanet.org/guidelines/testosterone-deficiency-guideline. Accessed 3 Nov 2020.

[31] Sharpless JL (2003). Polycystic ovary syndrome and the metabolic syndrome. Clin Diabetes.

[32] Barthelmess EK, Naz RK (2014). Polycystic ovary syndrome: current status and future perspective. Front Biosci Elite Ed.

[33] Goldman AL, Bhasin S, Wu FCW, Krishna M, Matsumoto AM, Jasuja R (2017). A reappraisal of testosterone’s binding in circulation: physiological and clinical implications. Endocr Rev.

[34] Vermeulen A, Verdonck L, Kaufman JM (1999). A critical evaluation of simple methods for the estimation of free testosterone in serum. J Clin Endocrinol Metab.

